# The effectiveness of PROLOTHERAPY for recalcitrant Medial TIBIAL Stress Syndrome: a prospective consecutive CASE series

**DOI:** 10.1186/s13047-021-00453-z

**Published:** 2021-04-16

**Authors:** Nat Padhiar, Mark Curtin, Osama Aweid, Bashaar Aweid, Dylan Morrissey, Otto Chan, Peter Malliaras, Tom Crisp

**Affiliations:** 1 Centre for Sports and Exercise Medicine, William Harvey Research Institute, Barts and The London School of Medicine and Dentistry, Queen Mary, University of London, London, UK; 2 London SportsCare, London Independent Hospital, London, UK; 3 European SportsCare, London, UK; 4 Monash University, Melbourne, Australia

**Keywords:** Prolotherapy, MTSS, Injection, Exercise-induced leg pain, Dextrose

## Abstract

**Background:**

Medial tibial stress syndrome (MTSS) is one of the most common lower leg injuries in sporting populations. It accounts for between 6 and 16% of all running injuries, and up to 53% of lower leg injuries in military recruits. Various treatment modalities are available with varying degrees of success. In recalcitrant cases, surgery is often the only option.

**Objective:**

To evaluate whether ultrasound-guided injection of 15% dextrose for treatment of recalcitrant MTSS decreases pain and facilitates a return to desired activity levels for those who may otherwise be considering surgery or giving up the sport.

**Method:**

The study design was a prospective consecutive case series involving eighteen patients: fifteen male and three female; (mean age = 31.2 years) with recalcitrant MTSS. They were referred from sports injury clinics across the UK, having failed all available conservative treatment.

**Intervention:**

An ultrasound-guided sub-periosteal injection of 15% dextrose was administered by the same clinician (NP) along the length of the symptomatic area. Typically, 1 mL of solution was injected per cm of the symptomatic area.

**Main outcome measures:**

Pain was assessed using a 10-cm visual analog scale (VAS) at baseline, short-term, medium-term (mean 18 weeks), and long-term (mean 52 weeks) follow-up. Symptom resolution and return to activity were measured using a Likert scale at medium and long-term follow-up. Statistical analyses were performed using SPSS for Mac version 19.0.0 (IBM, New York, NY, US). The Shapiro-Wilk test was used to evaluate the normality of the distribution of data. Friedman’s non-parametric test was used to compare the within-patient treatment response over time. *Post-hoc* Wilcoxon signed-rank tests with Bonferroni corrections were performed to determine VAS average pain response to treatment over five paired periods.

**Results:**

Patients reported a significant (*p* < 0.01) reduction in median VAS pain score at medium and long-term follow-up compared to baseline. Median improvement per patient was 4.5/10. Patients rated their condition as ‘much improved’ at medium-term follow-up and the median return to sports score was ‘returned to desired but not pre-injury level’ at medium-term and long-term follow-up. No adverse events were reported.

**Conclusions:**

Ultrasound-guided 15% dextrose prolotherapy injection has a significant medium-term effect on pain in MTSS. This benefit may be maintained long-term; however, more robust trials are required to validate these findings in the absence of controls.

**Clinical relevance:**

Clinicians should consider the use of ultrasound-guided injection of 15% dextrose as a viable treatment option to reduce pain and aid return to activity for patients with recalcitrant MTSS.

## Introduction

MTSS is one of the most common lower leg injuries in sporting populations [1]. It accounts for between 6 and 16% of all running injuries [2], and up to 53% of lower leg injuries in military recruits [3].

The diagnosis is reliably based on a detailed history and physical examination with pain provoked on palpation of the lower one-third of medial tibial [4].

In the early stages of the condition, pain tends to occur at the beginning of exercise, may diminish as activity proceeds, and recurs at the end [2, 5, 6]. Usually, the pain resolves over a variable period of rest [7]. However, as the condition progresses, pain may occur constantly throughout the exercise [8], at rest, and at night, causing significant distress and affecting the quality of life [2, 5, 9].

MTSS is predominantly managed conservatively. Treatment modalities that have shown potential benefit include extracorporeal shockwave therapy (ESWT), ultrasound therapy, iontophoresis, ice massage, periosteal pecking [10–13], non-steroidal anti-inflammatory drugs (NSAIDs), stretching and foot orthoses [14], and modification of biomechanical factors. However, a systematic review by Winters et al. [15] showed no evidence for the effect of any intervention in treating MTSS. In recalcitrant cases surgery is considered, although it significantly reduced pain in 72% of those treated, only 41% of patients returned to the sport at their previous level [16]. Although common, the pathophysiological process underlying MTSS remains uncertain. A popular theory is that excessive muscular traction could lead to inflammation of the periosteum, causing chronic periostitis [8, 17, 18]. However, histological studies have found scant evidence of inflammation [19–21]. More recently, it has been suggested that MTSS be classified as a point on a continuum of bone stress reaction, which can be assessed using magnetic resonance imaging (MRI) [22]. Beck (1998) proposed that repetitive loading during sustained weight-bearing activity may lead to strain-related periosteal remodeling due to tibial bending, which provokes stress injury at the point of maximum bending [23].

Winters et al. (2019) found linear microcracks in the biopsies of athletes with MTSS with no repair reaction, suggesting unrepaired microdamage as underlying pathophysiology [24].

Proliferative injection therapy (prolotherapy) has been used clinically since the late nineteenth century and has been mentioned in medical journals since at least 1937 [25]. The rationale behind prolotherapy is that injecting proliferants, such as hypertonic glucose solution, into damaged connective tissue, initiates inflammation, which leads to a healing cascade resulting in fibroplasia, deposition of new collagen and tissue hypertrophy [26] Animal studies have reported collagen proliferation, increased bone-ligament-bone junction strength and ligament mass with prolotherapy injections compared to controls [27]. The periosteum is richly innervated with nociceptive nerve fibers [28], therefore in MTSS, a prolotherapy injection may reduce pain by disrupting these sensory fibers as a result of the direct osmotic shock action of hypertonic dextrose on cells local to the injection site [26].

Trials of prolotherapy have found it to be beneficial in the treatment of lateral epicondylopathy [29], osteitis pubis [30], plantar fasciopathy [31], Achilles tendinopathy [32], and recalcitrant coccygodynia [33]. There is currently no published literature investigating the use of prolotherapy in the management of MTSS. A pilot study of this project reported a median pain reduction of 50% at medium-term follow-up [34]. The present study was a continuation of that pilot study and enabled longer-term treatment effects to be established. With current management options for recalcitrant MTSS showing inconsistent or unsatisfactory results, investigation of this novel treatment was necessary.

This prospective case series study sought to evaluate whether ultrasound-guided injection of hypertonic dextrose decreases pain and facilitates a return to desired activity levels for those who may otherwise be considering surgery or giving up the sport. We hypothesise that prolotherapy improves pain and facilitates a return to sport at the desired level.

## Patients and methods

### Recruitment

**Fig. 1:**
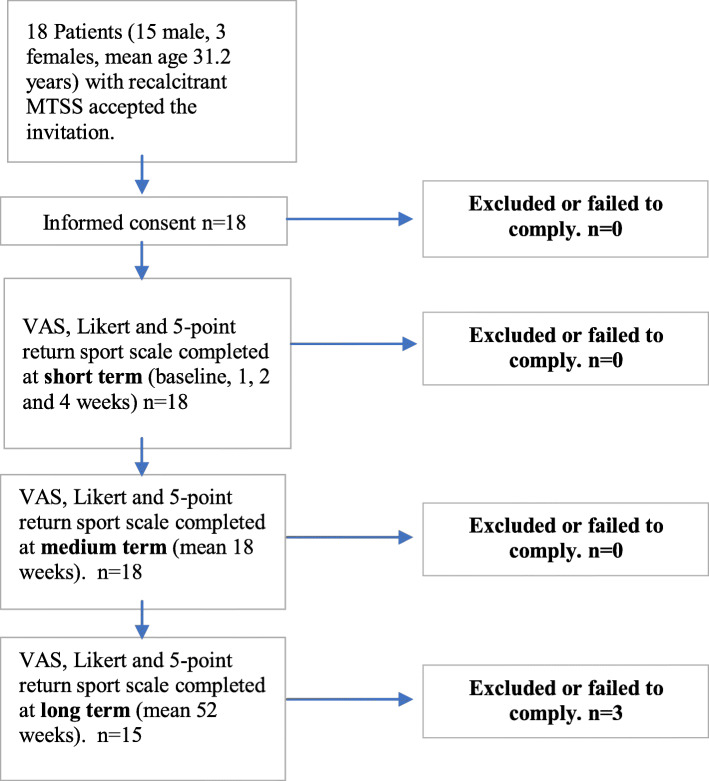
Recruitment process

Sports physicians, orthopaedic surgeons, podiatrists, podiatric surgeons, and physiotherapists working in sports injury clinics who were known to the main 2 authors (NP, TAC) across the UK were contacted via email and invited to refer patients with painful, recalcitrant MTSS that had not responded to other conservative treatment modalities, whom they felt might benefit from the trial intervention (Fig. 1.). The failed conservative treatments included, (a) rest, ice, compression, elevation (RICE), (b) assessing and addressing any lower limb functional factors (muscle strength & flexibility, proprioception & balance, (c) therapeutic ultrasound therapy, (d) ESWT, (e) acupuncture, (f) needling, (g) Graston fascia release, (h) improving neurodynamic, (i) non-steroidal anti-inflammatory drugs (NSAIDs), (j) correction of lower limb biomechanics (e.g., foot orthoses, brace, and taping for control of foot pronation), and (k) walking/running gait assessment and gait re-training.

Inclusion criteria were patients with persistent, painful MTSS assessed and confirmed by the lead clinician (NP). Exclusion criteria were previous periosteal surgery for MTSS, previous or current tibial stress fracture, or contraindications to the intervention such as pregnancy or anticoagulant therapy.

Patients were assessed by the lead clinician and the diagnosis of MTSS was confirmed by a history of exercise-induced pain over the posteromedial border of the middle to the distal third of the tibia, a positive Shin Palpation Test (palpation tenderness over the painful middle and distal thirds of the medial tibia) both at rest and following exercise, and MRI changes showing periosteal or bone marrow oedema.

Ethical approval was granted by the Queen Mary University of London Ethics Review Board (QMREC2009/22). Participants provided signed written informed consent. Subsequently, a detailed medical history and biographical data were taken and an examination was performed.

### Intervention and injection procedure

**Fig. 2:**
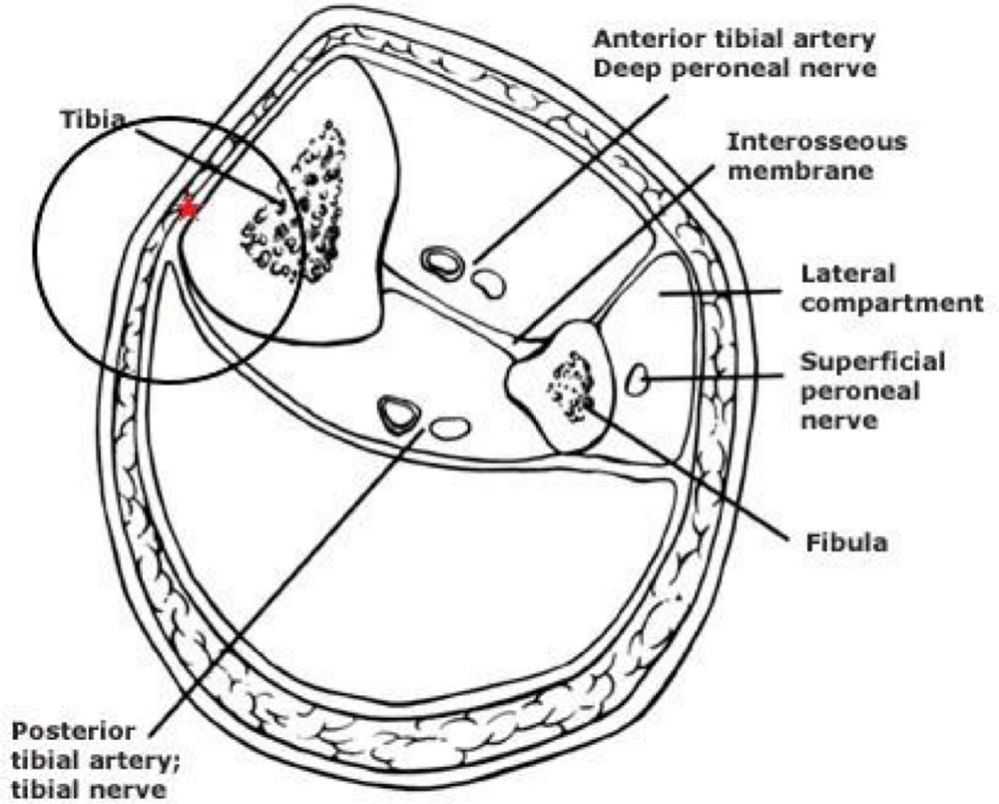
Cross section diagram of the leg. Red star marks the target area, anterior to deep crural fascia along the medial tibia

**Fig. 3:**
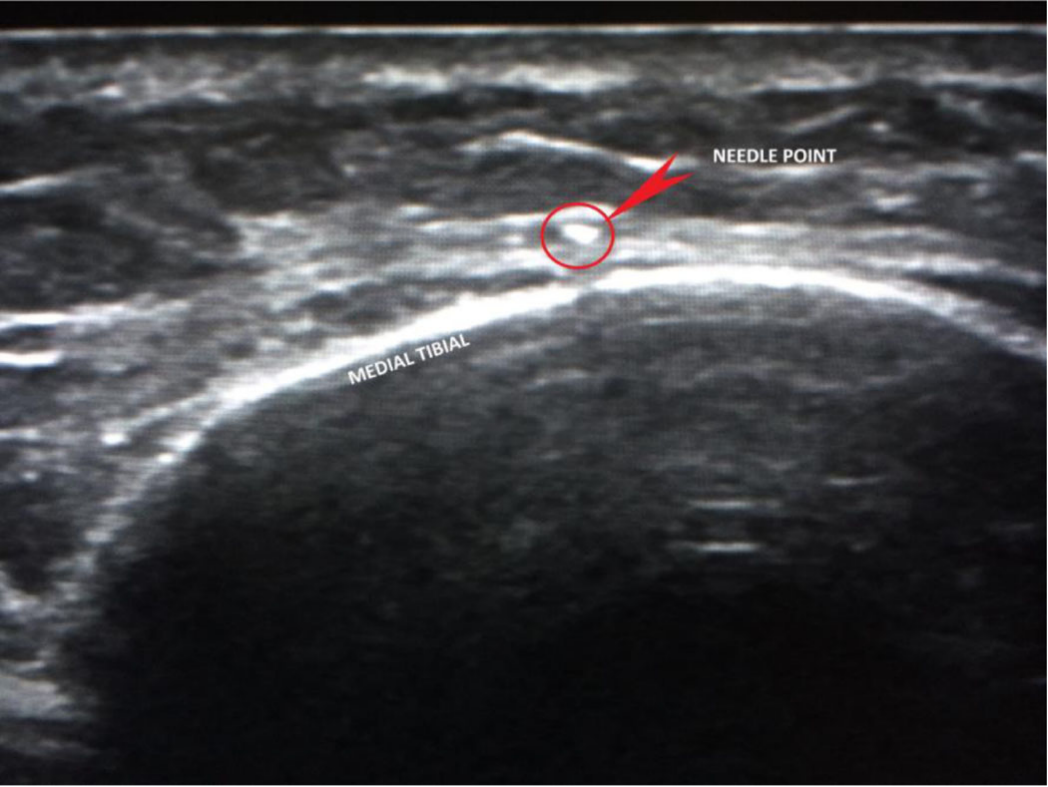
Transverse ultrasound image of the needle position (marked with the red circle/arrow)

**Fig. 4:**
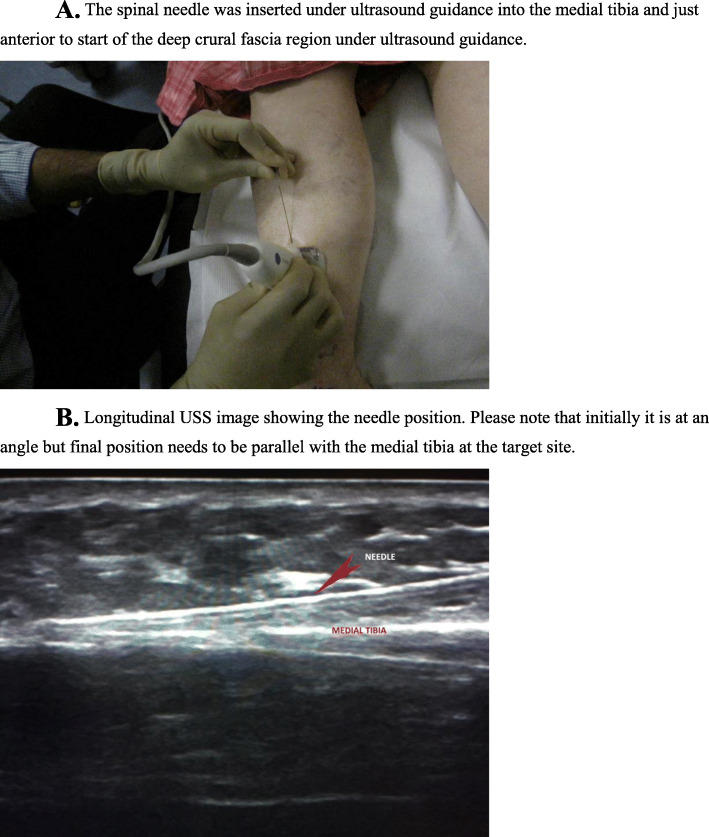
**A.** The spinal needle was inserted under ultrasound guidance into the medial tibia and just anterior to start of the deep crural fascia region under ultrasound guidance. **B.** Longitudinal USS image showing the needle position. Please note that initially it is at an angle but final position needs to be parallel with the medial tibia at the target site

**Fig. 5:**
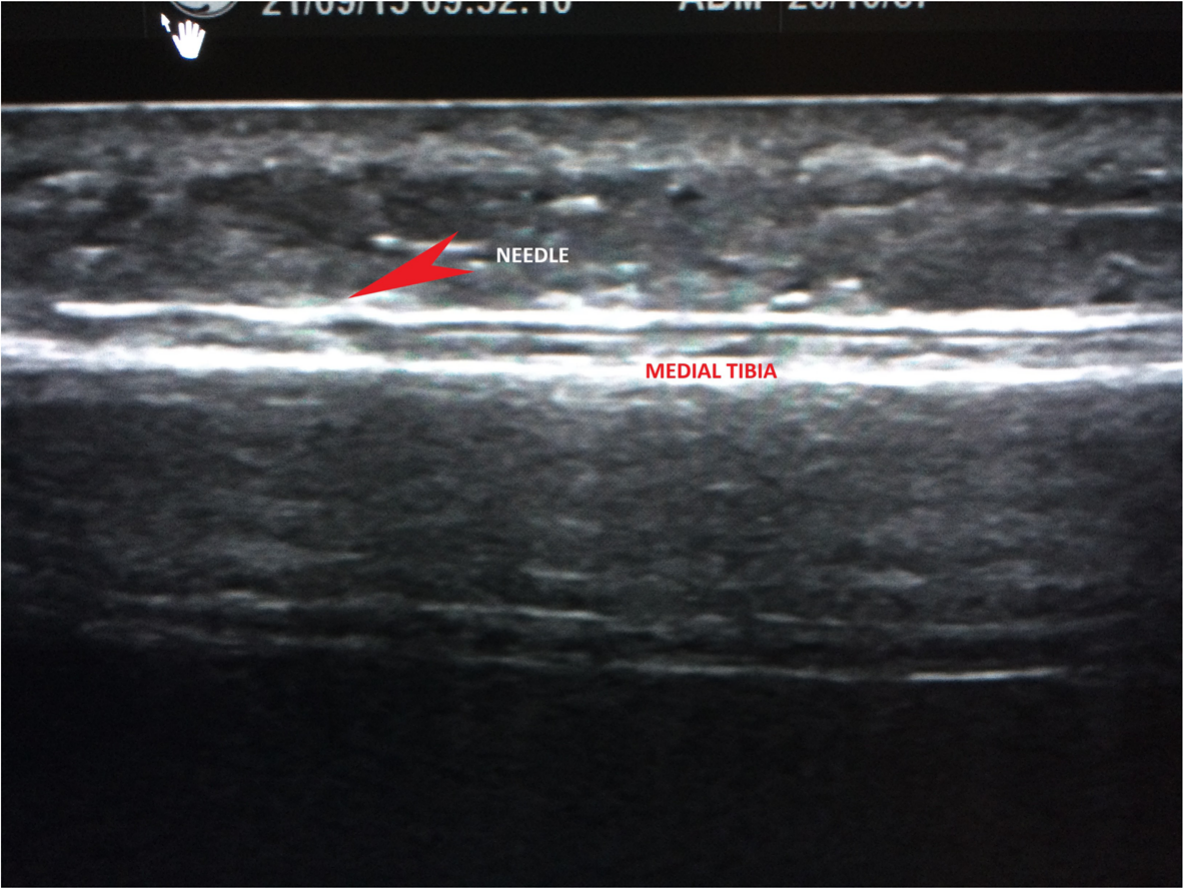
Longitudinal USS image showing the final needle position which is positioned parallel with the medial tibia at the target site

**Fig. 6:**
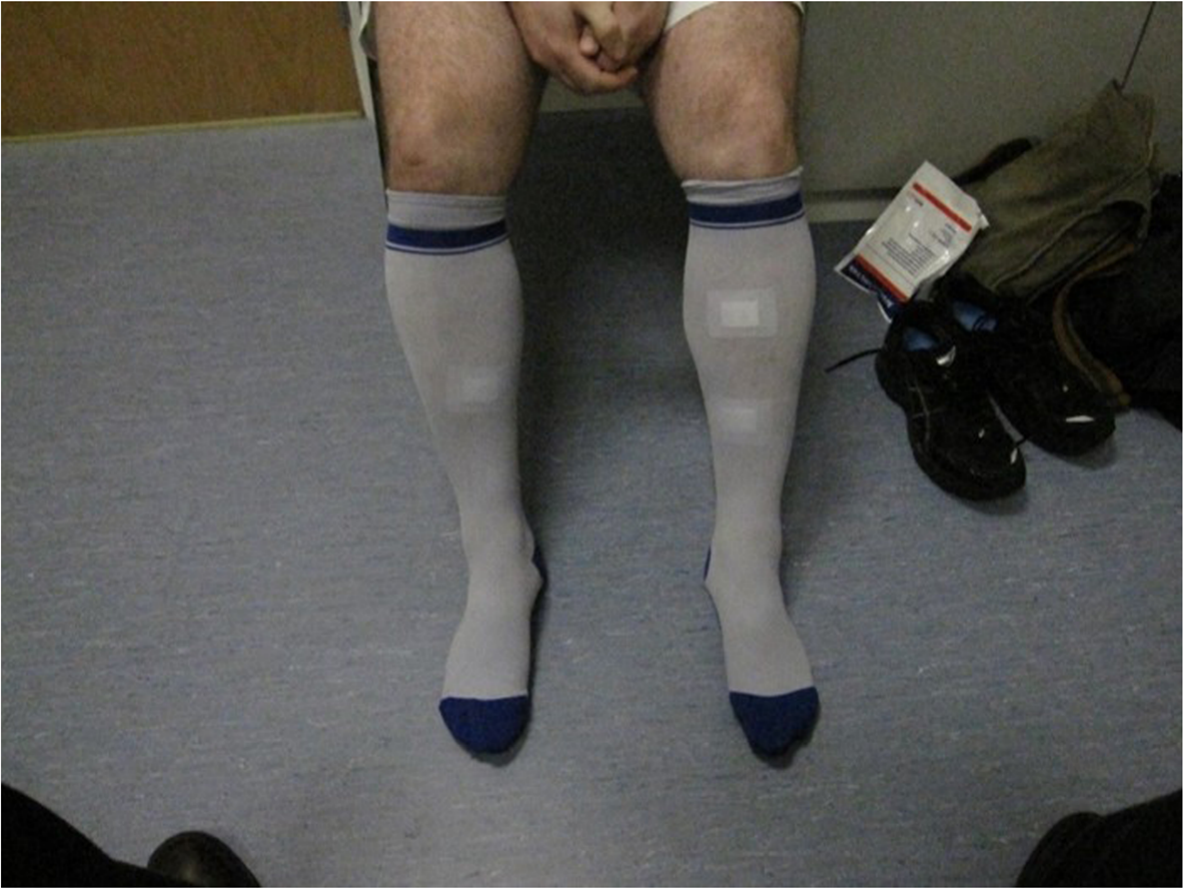
After the injection care involved cleansing the skin, applying wound dressing, ice and compression socks

The injection procedure is very simple and within the expertise of all clinicians who are used to injection techniques for musculoskeletal pathology. It is primarily performed in the out-patient department (OPD) and does not require a local anaesthetic even though local anaesthetic is used to dilute 50% glucose down to 15%. In some cases where pain tolerance is poor, it can be performed under a general anaesthetic. In this study, all subjects were treated in OPD. The target area for needle placement in the area anterior to the deep crural fascia (Figs. 2 and 3.) along the medial tibia. The skin overlying the most painful area of the tibia was marked with an indelible marker pen and then cleansed using alcoholic chlorhexidine (2% chlorhexidine gluconate, 70% isopropyl alcohol). An ultrasound scanner (USS) (Siemens AG, Berlin, Germany) was used to guide and confirm needle position. The needle is introduced under real-time USS from the most proximal end of the site of pain (knee end) to the most distal (ankle end) (Figs. 4a & b). The spinal needle (0.7 mm diameter × 90 mm length, Becton, Dickinson and Company LLC, Franklin Lakes, New Jersey, US) was positioned parallel along the medial tibia in the area anterior to the deep crural fascia (Fig. 5). The needle introducer was removed with the needle in place. 15% dextrose solution was slowly infiltrated, approximately 1 mL of solution per 1 cm along the whole length of the area of pain. In some cases where the length was longer than the spinal needle, the second entry point was made following the same protocol as above. After the injection, the area was cleaned, dressed, ice packs applied for 2 min, and knee high compression socks (Fig. 6.) (Bauerfeind AG, Zeulenroda-Triebes, Germany) were fitted. Patients were advised to continue wearing them for up to four weeks, removing them at night. Patients were advised to take relative rest for three days and advised for simple flexibility exercises and a graded return to physical activity. Patients were followed up one week later to monitor progress and address any concerns or questions. Patients were also given an emergency mobile number to call if they experienced any adverse reaction to the injection. This would include, extreme unrelenting pain, erythema, swelling, delayed allergic rash or sleep disturbance.

### Outcome measures

The patients’ ‘average pain’, defined as the most pervasive severity of pain throughout 24 h, was measured using a 10-cm visual analog scale (VAS). The VAS is sensitive [35], reliable, valid, and responsive for measuring pain in other common musculoskeletal conditions, such as patellofemoral pain syndrome [36]. The pain was assessed in this way at 0 (baseline), 1, 2, and 4 weeks after the injection, and at medium-term (mean 18 weeks, range 13–36 weeks) and long-term (mean 52 weeks, range 47–74 weeks) follow-up to assess the patients’ response to the intervention.

A Likert [37] symptom resolution scale was used to measure the subjective degree of recovery at medium-term and long-term follow-up compared to baseline. There are six possible outcome scores for the Likert scale: 1– completely recovered, 2– much improved, 3– somewhat improved, 4– no change, 5– worse, 6– much worse. Treatment was classed as a success in patients who rated themselves as ‘completely recovered’ or ‘much improved’, reflecting the method of previous authors [38]. Other scores were considered a treatment failure. Categorical scales such as the Likert are sensitive indicators of clinical trial endpoints [37].

Return to the sport was assessed with a five-point activity scale at medium-term and long-term follow-up: 1– not active at all, 2– no return to sport, 3– returned to the sport at an unsatisfactory lower level, 4– returned at desired but not pre-injury level, 5– returned at the pre-injury level. Although not reported in other literature, treatment aimed to enable patients to return to their desired sports at pre-injury levels. All other activity scores were considered a treatment failure.

### Statistical analysis

Statistical analyses were performed using SPSS for Mac version 19.0.0 (IBM, New York, NY, US). Statistical significance was set at a *p*-value less than 0.05. The Shapiro-Wilk test was used to evaluate the normality of the distribution of data. The distribution of data were negatively skewed and not normally distributed (Shapiro-Wilk = 0.009) therefore appropriate non-parametric tests were performed to evaluate the changes in pain levels.

Median values and interquartile ranges were calculated to compare baseline and follow-up data for VAS average pain scores, Likert symptom resolution, and return to sports scores.

Friedman’s non-parametric test was used to compare the within-patient treatment response over time. *Post-hoc* Wilcoxon signed-rank tests with Bonferroni corrections were performed to determine VAS average pain response to treatment over five paired periods (baseline – 4 weeks, baseline – 18 weeks, 4–18 weeks, baseline – 52 weeks, 4 weeks – 52 weeks). The Bonferroni corrected alpha value was (*p* < 0.01).

## Results

Twenty-five legs of eighteen patients were injected, of that twenty-five, seven legs were injected a second time. For data collection, each patient was treated as a whole case rather than individual legs.

On average, patients reported that post-injection pain took three days to settle. Side effects were not asked for specifically, but there were no self-reported adverse events following injection.

The mean age and duration of symptoms for the patients are summarised in Table 1. Two patients had undergone surgical fasciotomy for diagnosed chronic compartment syndrome on their affected limbs.

**Table 1:** Mean age and duration of symptoms

Characteristic	
Male	15 (83%)
Female	3 (17%)
Mean age	34 (SD 10.7)
Mean symptom duration (weeks)	52 (SD 9.1)
Mean BMI	25.9 (SD 3.4)

BMI = Body Mass Index

### Descriptive analysis

Data for eighteen patients were available for analysis of treatment effect to medium-term and fifteen patients to long-term follow-up. Three patients were lost to follow-up and these data points were not imputed but omitted from the long-term analysis.

**Fig. 7:**
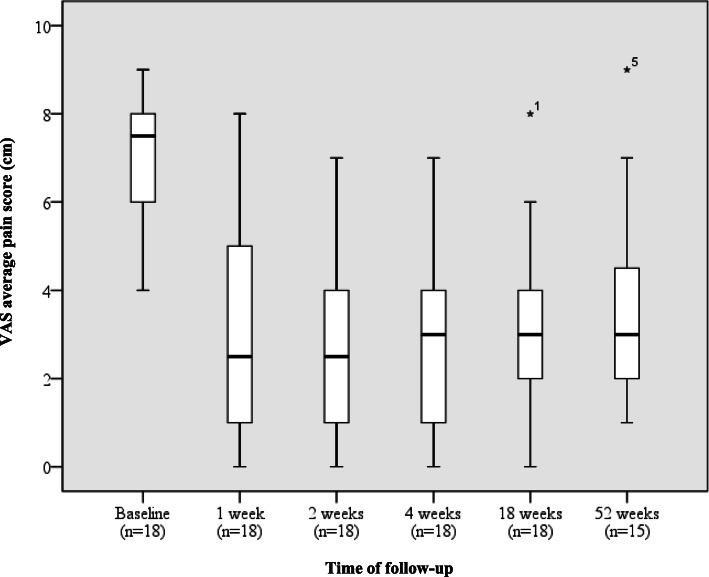
Box plot of median and interquartile range VAS average pain scores for the group of patients at follow-up. * Indicates a potential outlier (number refers to patient number)

Medians and interquartile ranges for the three outcome measures are displayed in Table.2. Change in median VAS average pain score over time is displayed in Fig. 7.

**Table 2:** Median values and interquartile ranges for VAS average pain, symptom resolution and level of activity

		Median	Interquartile range
**VAS average pain score (cm)** 0 – no pain 10 – worst pain imaginable	*Baseline*	7.5	6–8
	*2 weeks*	2	1–3.75
	*4 weeks*	3	1–4
	*18 weeks*	3	2–4
	*52 weeks*	3	2–4.5
**Likert symptom resolution score** 1 – completely recovered 6 – much worse	*18 weeks*	2	2–3
	*52 weeks*	3	2–4
**Activity level** 1 – not active at all 5 – active at pre-injury level	*18 weeks*	4	3–5
	*52 weeks*	4	3–4.5

There was complete resolution of symptoms at 18 weeks and 52 weeks in two patients (11%). There were much improved symptoms at 18 weeks in eight patients (33%) and 52 weeks in three patients (20%). At 18 weeks, six patients (33%) were somewhat improved and at 52 weeks the same improvement was observed in three patients (20%). There was no change in symptom resolution at 18 weeks in two patients (11%) and 52 weeks in seven patients (47%). There were no patients who were worse or made worse following prolotherapy (Table.3.).

**Table 3:** Summary of symptom resolution and activity level outcomes

	18 weeks (*n* = 18)	1 year (*n* = 15)
Likert symptom resolution		
Completely recovered	2 (11.11%)	2 (13.33%)
Much improved	8 (33.33%)	3 (20%)
Somewhat improved	6 (33.33%)	3 (20%)
No change	2 (11.11%)	7 (46.67%)
Worse	0 (0%)	0 (0%)
Much worse	0 (0%)	0 (0%)
Activity level		
Returned at pre-injury level	5 (27.78%)	4 (26.67%)
Returned at desired but not pre-injury level	5 (27.78%)	4 (26.67%)
Returned to sport at an unsatisfactory lower level	8 (44.44%)	6 (40%)
No return to sport	0 (0%)	1 (6.67%)
Not active at all	0 (0%)	0 (0%)

At 18 weeks, five patients (28%) returned to pre-injury level return to sport, and at 52 weeks four patients (27%). At 18 weeks, five patients (28%) returned to the desired but not pre-injury level return to sport, and at 52 weeks, four patients (27%). At 18 weeks, eight patients (44%) returned to the sport at an unsatisfactory level, and at 52 weeks, six patients (40%). One patient failed to return to any level of sport or activity and elected to undergo surgery (Table.3.).

### Statistical analysis

Friedman’s test of the VAS average pain scores to medium-term (mean 18 weeks) reported a Chi-squared value of 30.3, *p* < 0.001. There was a statistically significant difference between the mean pain ranks over time. Therefore, *a post-hoc* analysis was warranted. Friedman’s test was also significant to long-term follow-up, reporting a Chi-squared value of 27.5, *p* = < 0.001.

Changes in VAS average pain score rank and *p*-values for the five paired periods are reported in Table 4. VAS average pain decreased for 16 patients from baseline to both 4 weeks (*p* < 0.001) and 18 weeks follow-up (*p* < 0.001). One patient had an increase in pain at 4 weeks which subsequently decreased at 18 weeks follow-up and another patient reported no change at 4 weeks and 18 weeks. Between 4 weeks and 18 weeks after the injection, the pain increased in seven patients (38.8%), decreased in five (27.7%), and remained the same in six patients (33.3%) (*p* = 0.405). Between baseline and 52 weeks, pain decreased in 13 patients (86.7%), remained the same in one patient (6.7%), and increased in one patient (6.7%) (*p* = 0.001). Between 4 weeks and 52 weeks, pain decreased in five patients (33.3%), remained the same in four patients (26.7%), and increased in six patients (40%) (*p* = 0.322).

**Table 4:** Rank data and significance for the Wilcoxon signed-rank tests for VAS average pain. A negative rank represents an improvement in a patient’s pain over that time period. A positive rank represents worsening pain over that time period

Time period	Ranks	N	***p***-value
**Baseline – 4 weeks** (n = 18)	Negative ranks	16	< 0.001
	Positive ranks	1	
	Ties	1	
**Baseline – 18 weeks**	Negative ranks	16	< 0.001
(n = 18)	Positive ranks	0	
	Ties	2	
**4 weeks – 18 weeks**	Negative ranks	5	0.405
(n = 18)	Positive ranks	7	
	Ties	6	
**Baseline – 52 weeks**	Negative ranks	13	0.001
(n = 15)	Positive ranks	1	
	Ties	1	
**4 weeks – 52 weeks**	Negative ranks	5	0.322
(n = 15)	Positive ranks	6	
	Ties	4	

After Bonferroni corrections were applied, the change in VAS average pain from baseline to 4, 18, and 52 weeks follow-up were statistically significant (*p* < 0.01). The changes in VAS average pain from 4 to 18 weeks and 4 to 52 weeks were not significant (*p* > 0.01).

## Discussion

The pain was significantly reduced (p < 0.01) over short, medium, and long-term compared to baseline, with only two patients not reporting a reduction in VAS average pain over these periods (Table 4). The median VAS average pain score improved by 4.5 points at medium and long-term follow-up compared to baseline, equivalent to a 60% reduction in pain (Table 2.).

However, a wider range of pain scores was seen in the long-term (Fig. 5.), in addition to a larger *p*-value for average pain reduction, possibly due to the lower statistical power at this stage of follow-up. There was also a trend for improvement in pain to recede from 4 weeks post-injection, suggesting that pain control is most effective within the first month after administration. This is a potential window of opportunity to implement other conservative management options that could facilitate long-term pain control.

At 18 weeks (medium-term) follow-up, ten patients (55.5%) reported their MTSS as ‘completely recovered’ or ‘much improved’, indicating treatment success at this stage. Six patients (40%) fulfilled these criteria at 52 weeks follow-up, which signifies a potential longer-term decline in treatment effect (Table 4.).

Five patients (27.7%) reported a return to sport at pre-injury levels at medium-term follow-up and four patients (26.7%) at long-term follow-up. Treatment seems to have a limited effect on this outcome measure. However, ten patients (55.5%) were active at the desired level or more at medium-term follow-up and eight (53.3%) at long-term follow-up, which suggests we may have been too stringent in selecting the treatment success criteria for this outcome measure. One patient elected to undergo surgery, as at 52 weeks there was no improvement and failed to return to sport.

## Conclusion

Dextrose prolotherapy injection was well tolerated and significantly improved pain in the short term and, its effect was retained in the long term. Adequate symptom resolution and return to sport were achieved at medium-term follow-up and return to sport were maintained long-term. This prospective consecutive case study consisting of selective recalcitrant cases, therefore, suggests that prolotherapy has a significant effect on short, medium, and long-term pain reduction but in the absence of controls, a more robust study is required to show this benefit.

Prolotherapy is an effective treatment modality in the management of recalcitrant MTSS and even though one patient elected to undergo surgery, this study was not designed to assess whether there will be a reduction in the number of patients that may require surgery. This study did not involve any histological sampling to explain the effect on the tissue. We postulate that 15% glucose acts as an osmotic and chemical irritant (dehydrating cells) along with damage to tissue through needling causing local trauma and bleeding. This provokes a cascade of inflammation, proliferation, and re-modeling. The rationale behind prolotherapy is that injecting proliferants, such as hypertonic glucose solution, into damaged connective tissue, initiates inflammation, which leads to a healing cascade resulting in fibroplasia, deposition of new collagen, and tissue hypertrophy [26]. It is also possible that prolotherapy improves the mechanical advantage with improved stability of deep crural fascia junction with the medial tibia. Animal studies have reported collagen proliferation, increased bone-ligament-bone junction strength, and ligament mass with prolotherapy injections compared to controls [27]. The reduction in pain may be due periosteum being richly innervated with nociceptive nerve fibers [28], therefore, in MTSS a prolotherapy injection may reduce pain by disrupting these sensory fibers as a result of the direct osmotic shock action of hypertonic dextrose on cells local to the injection site [26]. It is also worth debating, even though there is no evidence, whether and if, the sensory fibers are disrupted, could this, apart from the positive effects on pain and function short-term, produce adverse effects long term e.g., disturbed bone remodeling potentially leading to stress fractures/worsening MTSS/osteoporosis?

However, in sport, the benefit of a pain-free phase can be effectively used to provide a platform for early rehabilitation and a window of opportunity to plan a return to sport or physical activity-specific rehabilitation.

## Limitations

As the first study investigating this novel injection technique, the use of a non-blinded, consecutive case series design without randomisation or a control group was a pragmatic choice given the difficulties of recruiting participants. Evidence of treatment effect is therefore limited. Despite results showing a significant treatment effect on pain, the mechanism by which it exerts this effect cannot be determined, and therefore the possibility of a placebo effect cannot be excluded in addition to the possibility these patients may have improved with time without treatment.

Patients were only included in the study if they had MRI evidence of periosteal changes or bone marrow oedema. MTSS is a clinical pain syndrome with unknown aetiology. The evidence suggests that periosteal and bone marrow oedema are absent in the majority (i.e., 56%) of clinically diagnosed cases with MTSS [39]. Furthermore, periosteal and bone marrow oedema is often present in healthy asymptomatic athletes [40, 41] which suggests it is not an adequate characteristic to identify those with MTSS. As a result, we may have missed relevant cases with MTSS with negative MRI findings.

Three patients were also lost to long term follow up, despite every effort to contact them to complete the study. In a consecutive case series of 18 subjects, this was a great loss.

Given that there were no validated scales to specifically assess treatment outcomes in EILP patients at the time of this study, we adapted Likert scales to measure symptom resolution and return to sport. Although Likert scales are sensitive indicators of clinical trial endpoints [37], the lack of specificity could at least partly explain the limited treatment effect for these outcomes. Since this study was conducted there are 3 validated patient outcome scores are now available [42–44], one is specific for MTSS [42].

## Context

The current literature on the effects of prolotherapy on chronic musculoskeletal conditions (lateral epicondylopathy, Achilles and other tendinopathies, osteitis pubis, plantar fasciopathy, recalcitrant coccygodynia, and osteoarthritis) is limited. Two case-series investigating the efficacy of dextrose prolotherapy injections at reducing pain in lateral epicondylitis [29] and chronic groin pain (osteitis pubis and/or adductor tendinopathy) [30] reported mean reductions of 5.3 and 5.0 respectively on 10-cm VAS pain scales. A double-blind randomised controlled trial reported a significant mean pain decrease of 4.6 on a 10-cm pain scale at 16 weeks from baseline [28]. Pain change in the control group was not significant. These results are comparable to our study, where a 4.5-point reduction of pain was reported at 18 weeks follow-up. One study observed patients to long-term follow-up (mean 11.8 months), reporting a mean VAS pain reduction of 5.3 points compared to baseline [30]. Pain reduction was maintained long-term in Ryan et al*’s* study [31], which is similar to the 4-point VAS average pain reduction in our study.

## Future studies

Future studies require more robust methodologies including larger participant numbers, a control group or crossover design, randomisation, and, if possible, blinding to improve the validity of the results. Adverse effects should be explicitly sought to permit a more thorough treatment profile to be compiled.

VAS average pain data from follow-up and one and two weeks post-injection appear of limited application regarding treatment direction, with the decision to re-inject patients with the suboptimal symptomatic response coming at four weeks or later. Follow-up at four weeks then monthly may allow better monitoring of the treatment response over the medium- to long-term and facilitate the decision to re-inject for those patients whose symptoms have not responded as well as predicted.

With some patients requesting a second injection, it may be prudent to administer more than one prolotherapy injection per symptomatic leg, reflecting the protocols of other studies where injections were administered weekly or monthly, ranging from 2 to 12 injections over the study period [29, 32, 33], or until complete resolution of symptoms or no improvements were seen [30, 32].

## Data Availability

There are no issues concerning this as strict confidentiality was observed and subjects were anonymised. The authors affirm that this manuscript is an honest, accurate, and transparent account of the study being reported; that no important aspects of the study have been omitted; and that any discrepancies from the study as planned (and, if relevant, registered) have been explained.
